# Where are we in 2021 with heart failure with reduced ejection fraction?—current outlook and expectations from new promising clinical trials

**DOI:** 10.1007/s10741-021-10120-x

**Published:** 2021-05-29

**Authors:** Agnieszka Dębska-Kozłowska, Marcin Książczyk, Małgorzata Lelonek

**Affiliations:** 1 Military Medical Academy Memorial Teaching Hospital, Central Veteran Hospital, Medical University of Lodz, Lodz, Poland; 2 Department of Noninvasive Cardiology, Medical University of Lodz, Lodz, Poland

**Keywords:** Chronic heart failure with reduced ejection fraction, Angiotensin receptor/neprilysin inhibitor, Sodium-glucose cotransporter inhibitors, Omecamtiv mecarbil, Vericiguat

## Abstract

Guideline-directed optimal medical therapy is a well-established therapy in treating patients with heart failure with reduced ejection fraction (HFrEF). Despite clear recommendations, the prognosis in this group of patients is still poor with high mortality. After publishing results of the PARADIGM-HF trial (Prospective Comparison of ARNI—Angiotensin Receptor/Neprilysin Inhibitors—with ACEI—Angiotensin-Converting Enzyme Inhibitor—to Determine Impact on Global Mortality and Morbidity in Heart Failure) clinical investigators accelerated their research. Recently, many new trials have been designed to evaluate the efficacy and safety of promising management, taking into account heterogeneity of population with chronic HFrEF. Determining target doses still poses the biggest problem in standard pharmacotherapy. Implementation of new substances for the HFrEF therapy makes it possible to formulate simple rules of treatment—in most cases, administering a dose of drug in one tablet provides a faster therapeutic effect. The aim of this article is to summarize current knowledge on recently announced findings on novel molecules and to propose a new revolutionary and individualised approach to treatment of HFrEF patients.

## Introduction

**Fig. 1:**
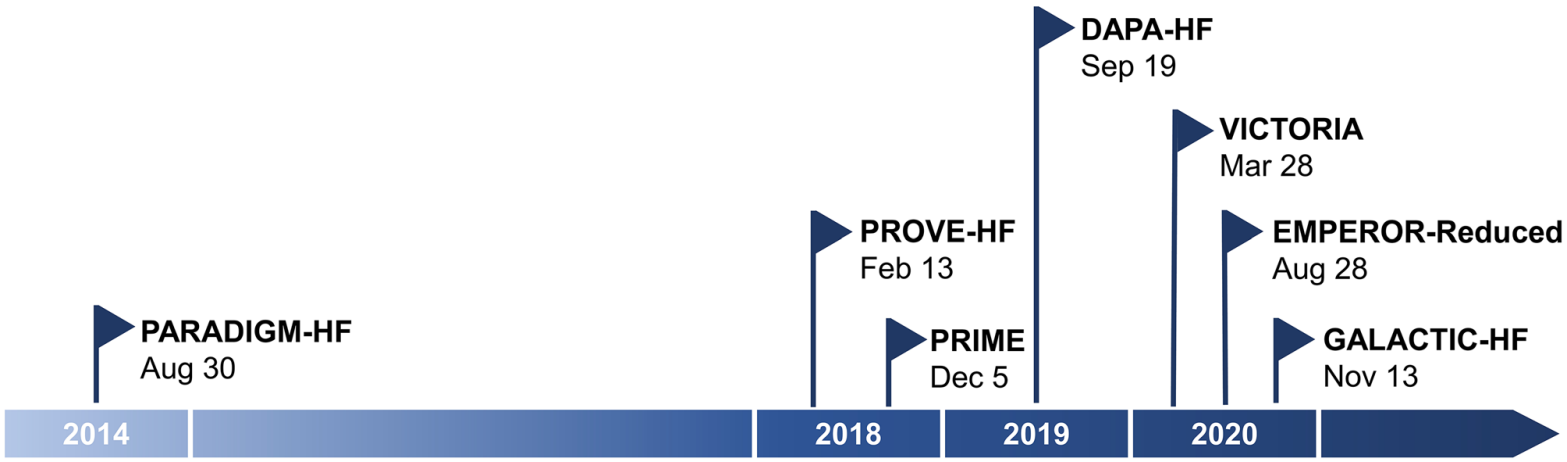
Historical timeline of breakthrough clinical trials for heart failure with reduced ejection fraction

Guideline-directed optimal medical therapy is a well-established therapy in treating patients with heart failure with reduced ejection fraction (HFrEF). Despite clear recommendations, the prognosis in this group of patients is still poor and characterized with high mortality. Due to an increasing worldwide burden, heart failure is a grave public health problem [1]. There is a need to focus attention on the earlier diagnosis and appropriate intervention for heart failure. After publishing results of the PARADIGM-HF trial (Prospective Comparison of ARNI—Angiotensin Receptor/Neprilysin Inhibitors—with ACEI—Angiotensin-Converting Enzyme Inhibitor—to Determine Impact on Global Mortality and Morbidity in Heart Failure) in the year 2014, clinical investigators accelerated their research (Fig. 1).

Many new trials have been recently designed to evaluate the efficacy and safety of a promising novel class of molecules, taking into account the heterogeneity of the population with chronic HFrEF. Their findings are invaluable as they enable to implement individualized therapies for HFrEF patients. Moreover, results of current research seem promising and revolutionary enough to be considered the grounds for future strong recommendations regarding treatment of HFrEF patients.

## First and foremost—angiotensin receptor/neprilysin inhibitors (ARNI)—a standard in HFrEF treatment

It is several years since results of the PARADIGM-HF trial were published in the New England Journal of Medicine [2]. The trial proved an overwhelming, sustained advantage and superiority of ARNI in reducing the risks of cardiovascular death or death due to other causes (hazard ratio, HR, 0.80; 95% confidence interval [CI], 0.71 to 0.89; *P* < 0.001 and HR, 0.84; 95% CI, 0.76 to 0.93; *P* < 0.001 respectively) and hospitalization for worsening heart failure (HR, 0.79; 95% CI, 0.71 to 0.89; *P* < 0.001) [2, 3]. These impressive results are undoubtedly considered a breakthrough in HFrEF treatment, which was reflected in the last 2016 guidelines on management of acute and chronic heart failure patients according to the European Society of Cardiology [4].

**Fig. 2:**
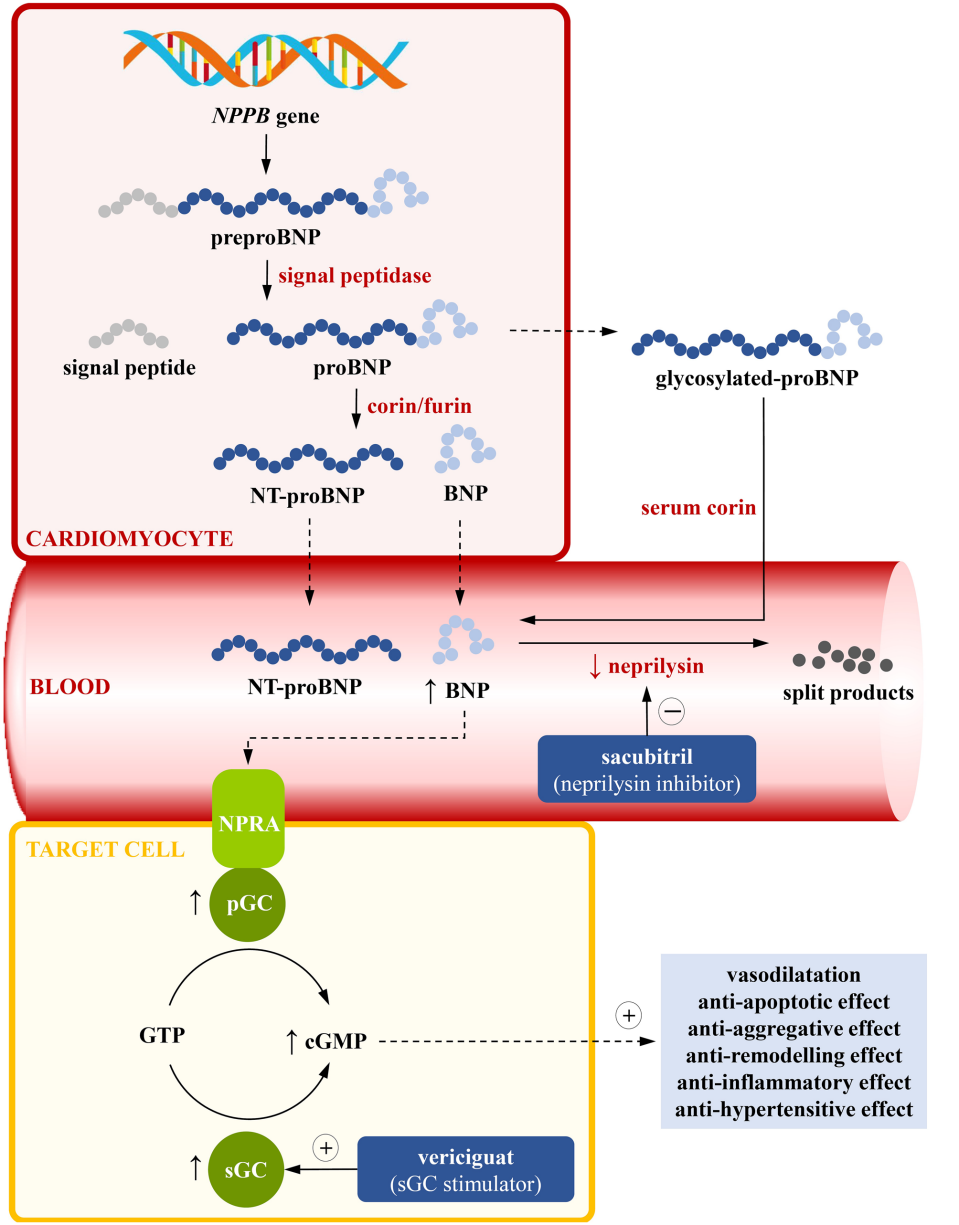
Pathways of synthesis and release of B-type natriuretic peptide and a pivotal role of cyclic guanosine monophosphate pathway modulation with sacubitril and vericiguat in heart failure outcome [9–11]. BNP B-type natriuretic peptide, cGMP cyclic guanosine monophosphate, GC guanyl cyclase, GTP guanosine triphosphate, NPPB natriuretic peptide B, NPRA natriuretic peptide receptor A, NT-proBNP N-terminal prohormone of BNP, pGC particulate guanylate cyclase, sGC soluble guanylate cyclase

The mechanism of action of sacubitril/valsartan (ARNI) is shown on Fig. 2 [5–7]. Natriuretic peptides (NPs) cause vasodilation by increasing production of cyclic guanosine monophosphate (cGMP) with subsequent reduction of cardiac preload and afterload [7]. It should be noted that the cGMP pathway modulation is common for sacubitril and vericiguat. Moreover, both ARB (angiotensin receptor blocker) and NP show anti-inflammatory, anti-fibrotic and anti-hypertrophic effects. Interference with the cGMP pathway becomes a remarkable pathophysiological target in patients with HFrEF. A combination of two strategies—inhibition of breakdown of NP and blockade of the renin angiotensin aldosterone system (RAAS)—turned out to be most beneficial in HFrEF patients [8].

Over the years, we have been gaining experience in the implementation of ARNI. The position of ARNI as a first-line agent in HFrEF treatment has become well established in real-life and recent studies. Importantly, its beneficial impact on cardiac remodelling has been confirmed. In PROVE-HF trial (Rationale and Methods of the Prospective Study of Biomarkers, Symptom Improvement, and Ventricular Remodeling During Sacubitril/Valsartan Therapy for Heart Failure), the principal end point was to determine a relationship between the changes in concentration of NT-proBNP and cardiac remodelling within 12 months [12]. In this study, the left ventricle (LV) function, LV volume and selected parameters of diastolic dysfunction were evaluated. After a definite time limit of the follow-up, the change in log2-NT-proBNP concentration was correlated with changes in LVEF (*r* = −0.381; *P* < 0.001); LV end-diastolic volume index, LVEDVI (*r* = 0.320; *P* < 0.001) and LV end-systolic volume index, LVESVI (*r* = 0.405; *P* < 0.001). LVEF increased from median 28.2 to 37.8% (difference, 9.4% [95% CI, 8.8% to 9.9%]; *P* < 0.001), and LV indexed volumes reduced respectively [13]. Moreover, significant decrease of the ratio of early transmitral Doppler velocity/early diastolic annular velocity (E/e′) and left atrial volume index (LAVI) was revealed in 12 months, which confirms a decrease in left atrial pressure.

The second study on the role of ARNI on reverse remodelling—the PRIME trial (Pharmacological Reduction of Functional, Ischemic Mitral Regurgitation)—confirmed that sacubitril/valsartan was superior in reducing functional mitral regurgitation (FMR) in comparison to valsartan applied alone in the standard therapy in the study group of 118 participants in NYHA II-III, with EF of 25 to 50% and significant FMR lasting > 6 months [14]. An improvement of FMR was mainly identified by a change in the effective regurgitant orifice area (EROA) of FMR within 12-month follow-up. It was demonstrated that the decrease in EROA was substantially greater in the sacubitril/valsartan group than in the valsartan group (−0.058 ± 0.095 vs. −0.018 ± 0.105 cm^2^; *P* = 0.032). Moreover, the reduction of EROA was correlated with a decrease in LV end-systolic volume, LVESV (*r* = 0.70, *P* < 0.001), or end-diastolic volume, LVEDV (*r* = 0.66, *P* < 0.001), in the sacubitril/valsartan group, and the valsartan group as well (LVESV: *r* = 0.67, *P* < 0.001; and LVEDV: *r* = 0.58, *P* < 0.001). The results about a regurgitant volume, being a component of secondary end-points assessments, were also in favour of the sacubitril/valsartan group compared with the valsartan group (mean difference, −7.3 mL; 95% CI, −12.6 to −1.9; *P* = 0.009). Besides, the decrease in parameters of diastolic LV function—E/e′ was significantly greater in the sacubitril/valsartan group than in the comparator group (mean difference of change, –2.7; 95% CI, –5.1 to –0.2; *P* = 0.037).

These findings confirm the role of sacubitril/valsartan in improvement of cardiac reverse remodelling.

## This is time for sodium-glucose co-transporter inhibitors—SGLT2—inhibitors in heart failure patients

**Fig. 3:**
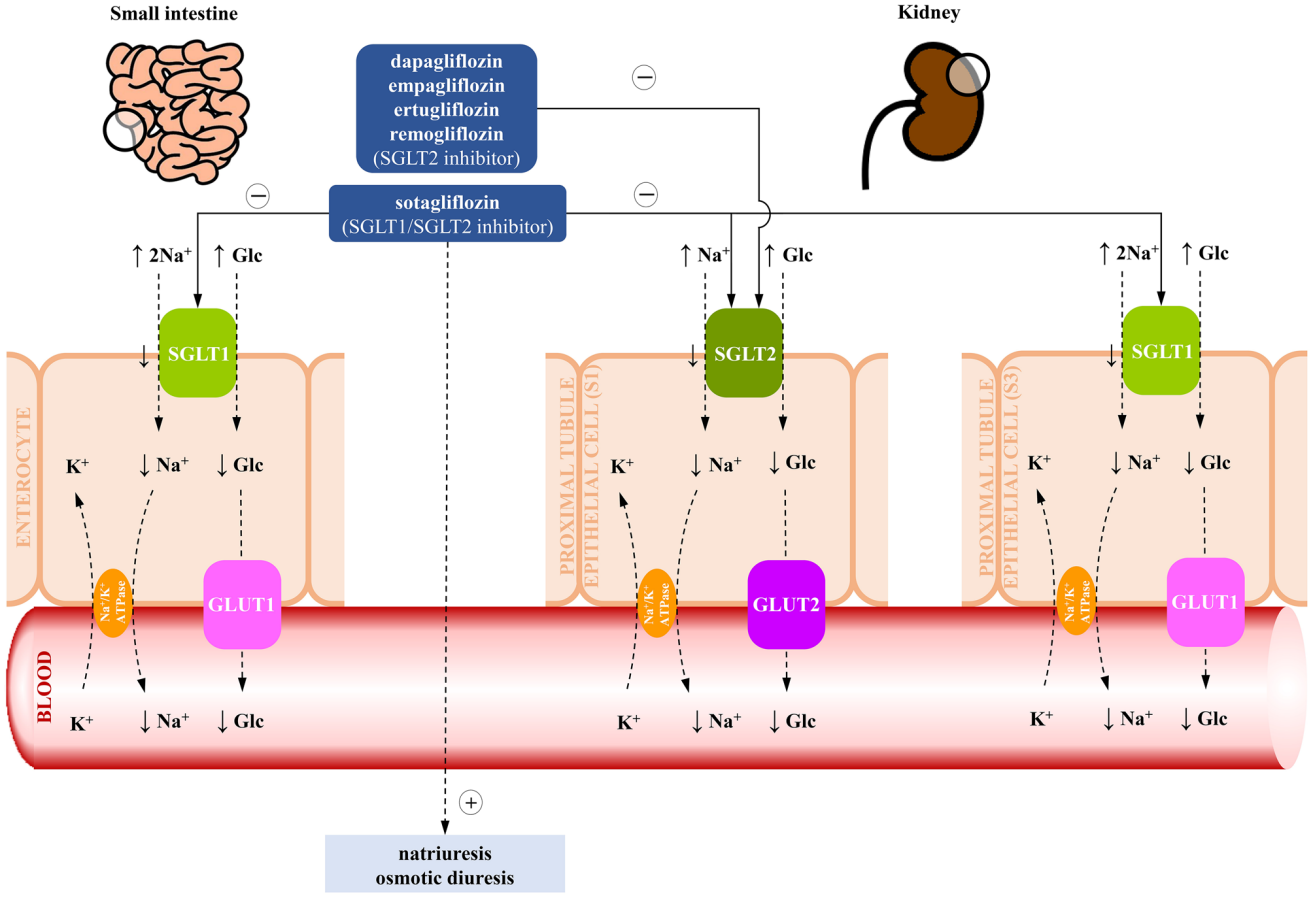
Mechanism of action of sodium‐glucose co‐transporter inhibitors [16, 17]. Glc glucose, GLUT1 glucose transporter 1, GLUT2 glucose transporter 2, K^+^ potassium, Na^+^ sodium, SGLT1 sodium‐glucose co‐transporter 1, SGLT2 sodium‐glucose co‐transporter 2

SGLT2 inhibitors (SGLT2i) are antidiabetic agents, which reduce glucose levels by promoting urinary glucose excretion with increasing natriuresis and osmotic diuresis [15] (Fig. 3).

Over the past few years, CV outcome trials have proved the pleiotropic action of SGLT2i. Positive findings of SGLT2i trials inspired investigators. A highly important question of whether SGLT2i is beneficial in HFrEF arises.

The randomized study with dapagliflozin, DAPA-HF (Dapagliflozin And Prevention of Adverse-outcomes in Heart Failure trial), was designed in HFrEF population regardless of the presence or absence of diabetes [18]. The main purpose of this study was to check the effect of dapagliflozin added in one daily dose of 10 mg to recommended optimal treatment of HFrEF on the occurrence of primary end-point, composed of a worsening heart failure (hospitalization/an urgent heart failure visit) or cardiovascular death compared with placebo. In a median 18.2-month follow-up, the group treated with dapagliflozin demonstrated better results than that administered placebo in each evaluated component [19]. The detailed results were as follows: the primary outcome was noticed in 16.3% individuals (386 of 2373 patients) as compared to 21.2% (502 of 2371 patients) in the placebo group (HR, 0.74; 95% CI, 0.65–0.85; *P* < 0.001). The risk of the first worsening heart failure event and death from cardiovascular causes or from any cause was lower in the dapagliflozin group (HR, 0.70; 95% CI, 0.59–0.83 and HR, 0.82; 95% CI, 0.69–0.98 and HR, 0.83; 95% CI, 0.71–0.97, respectively). What is worth emphasizing is the fact that the findings were similar in diabetic and non-diabetic patients with HFrEF just after randomization, and beneficial clinical effect was observed after 28 days. Unlike empagliflozin in the EMPEROR-Reduced study (Empagliflozin Outcome Trial in Patients With Chronic Heart Failure with Reduced Ejection Fraction), dapagliflozin improved the physical function and quality of life in the studied population. The ground-breaking results of DAPA-HF resulted in the approval of dapagliflozin by the US Food and Drug Administration in May 2020 for treatment of patients with HFrEF [20].

Packer M. et al. analysed a group of 3730 patients with EF 40% or less in II-IV NYHA, randomly assigned to receiving 10 mg of empagliflozin or placebo in the EMPEROR-Reduced trial [21]. The study population of EMPEROR-Reduced had a lower LVEF, a higher concentration of NT-proBNP and was better treated (19% ARNI, 43% devices), in comparison with those enrolled in the DAPA-HF trial (Table 1). An analysis of rates of the composite end-point—cardiovascular death or hospitalization for worsening heart failure—revealed that those treated with empagliflozin were at significantly lower risk (HR, 0.75; 95% CI, 0.65–0.86; *P* < 0.001) regardless if they were patients with concurrent T2D or without during a median 16-month follow-up, whereas the impact of empagliflozin on CV death reduction and all-cause mortality alone was insignificant (HR, 0.92; 95% CI, 0.75–1.12 and HR 0.92; 95% CI, 0.77–1.10, respectively). Considering the risk of worsening HF events, statistically significant beneficial effects were seen very early after randomization, i.e. within 12 to 28 days following the initiation of treatment [22].

**Table 1:** Comparison of breakthrough studies for heart failure with reduced ejection fraction

Characteristic	PARADIGM-HF	DAPA-HF	VICTORIA	EMPEROR-Reduced	GALACTIC-HF
IND *vs* comparator/placebo	sacubitril/valsartan *vs* enalapril	dapagliflozin *vs* placebo	vericiguat *vs* placebo	empagliflozin *vs* placebo	omecamtiv mecarbil *vs* placebo
*n *	8442	4744	5050	3730	8232
Female – no (%)	21	23	24	24	21
Mean age (years)	64	66	67	67	65
LVEF (%)	29	31	29	27	27
NYHA class – no (%)	I (4.6) II (70.1) III (23.0) IV (0.7)	II (67.5) III (31.6) IV (0.9)	I (0.1) II (59.0) III (39.7) IV (1.3)	II (75.0) III (24.4) IV (0.6)	II (53.1) III (43.9) IV (3.0)
Median NT-proBNP (pg/ml)	1615	1437	2816	1906	2001
Ischemic heart failure – no (%)	60	56	58	52	54
ARNI – no (%)	n/a	11	15	19	19
ICD – no (%)	15	26	28	31	32
CRT – no (%)	7	7	15	12	14
eGFR 30-59 ml/min. – no (%)	33	41	43	43	46
eGFR <30 ml/min. – no (%)	─	─	10	6	6
Current hospitalization for heart failure or urgent visit to emergency department – no (%)	─	─	─	─	25
Hospitalization for heart failure in previous 3 months – no (%)	─	─	67	─	─
Hospitalization for heart failure in previous 3–6 months – no (%)	─	─	17	─	─
Hospitalization for heart failure in previous 12 months – no (%)	63	47	─	31	75
Primary outcome	composite of death from CV causes or a first hospitalization for HF	composite of death from CV causes or worsening HF (hospitalization or an urgent visit resulting in implementation of intravenous therapy for HF)	composite of death from CV causes or a first hospitalization for HF	composite of death from CV causes or a hospitalization for HF	composite of death from CV causes or a first HF event (an urgent clinic visit, emergency department visit, or hospitalization due to worsening HF, resulting in treatment intensification without a change in oral diuretic therapy)
Secondary outcomes	• death from any cause, •change from baseline to 8 months in the clinical summary score on the KCCQ, •time to a new onset of AF, •time to the first occurrence of a decline in the renal function	• composite of hospitalization for HF or CV death, • total number of hospitalizations for HF and CV deaths, •change from baseline to 8 months in the KCCQ, • composite of worsening renal function or renal death, •death from any cause	• death from CV causes, • first hospitalization for HF, • first and subsequent hospitalizations for HF, • composite of death from any cause or first hospitalization for HF, • death from any cause	• all hospitalizations for HF, • rate of the decline in the eGFR	• CV death, • change from baseline to week 24 in the clinical summary score on the KCCQ, • first HF hospitalization, • death from any cause
NNT for the CV death or HF hospitalization	22/2.25 years	21/1.5 years	34/10.8 months	19/1.3 years	No data available

*ARNI* angiotensin receptor/neprilysin inhibitor, *CRT* cardiac resynchronization therapy, *CV* cardiovascular, *eGFR *estimated glomerular filtration rate, *HF* heart failure, *ICD* implantable cardioverter-defibrillator, *IND* investigational new drug, *KCCQ* Kansas City Cardiomyopathy Questionnaire, *LVEF* left ventricle ejection fraction, *NNT* number needed to treat, *NT-proBNP* N-terminal prohormone for type B natriuretic peptide, *NYHA* New York Heart Association, *PARADIGM-HF* Prospective Comparison of ARNI with ACEI to Determine Impact on Global Mortality and Morbidity in Heart Failure Trial, *DAPA-HF* Dapagliflozin and Prevention of Adverse Outcomes in Heart Failure, *VICTORIA* Vericiguat Global Study in Subjects with Heart Failure with Reduced Ejection Fraction, *EMPEROR-Reduced* Empagliflozin Outcome Trial in Patients with Chronic Heart Failure and a Reduced Ejection Fraction, *GALACTIC-HF* Global Approach to Lowering Adverse Cardiac Outcomes through Improving Contractility in Heart Failure

The study population of EMPEROR-Reduced was also categorized by Zannad et al. [23] according to presence or absence of chronic kidney disease (CKD) defined at baseline as estimated glomerular filtration rate (eGFR) < 60 ml/min/1.73 m^2^ or albumin-to-creatine ratio > 300 mg/g (more than a half of the enrolled participants had CKD). The aim of the study was to evaluate the occurrence of the following end-points in both subgroups in a median 16-month observation: (1) a composite of CV death or HF hospitalization (HHF) as a primary outcome, (2) total HHF and (3) eGFR decrease. It has been shown that use of empagliflozin substantially reduces the incidence of the primary end-point and total HHF in patients with and without CKD—HR, 0.78; 95% CI, 0.65–0.93 and HR, 0.72; 95% CI, 0.58–0.90, respectively (interaction *P* = 0.63). Empagliflozin slowed the progression of CKD, which was manifested by a decreased level of eGFR by 1.11 ml/min/1.73 m^2^/year (0.23–1.98) in patients with CKD and by 2.41 ml/min/1.73 m^2^/year (1.49–3.32) in patients without CKD (interaction *P* = 0.045). Moreover, the outcome of exploratory composite kidney, comprising sustained profound decline in eGFR, chronic dialysis or kidney transplant was observed similarly in study subgroups—in those with and without CKD (HR, 0.53; 95% CI, 0.31–0.91 and HR, 0.46; 95% CI, 0.22–0.99, respectively; interaction *P* = 0.78).

What are the implications for daily clinical practice? Empagliflozin is well tolerated in CKD patients and reduces serious CV outcomes including death and hospitalization in HFrEF patients with and without CKD, even in those with advanced impairment of renal failure, manifested by eGFR as low as 20 mL/min/1.73 m^2^.

Recent SGLT2i trials documented benefits of various HFrEF patients. Moreover, the benefit on microvascular, in particular renal outcome and nephroprotective effects, was also strongly documented in HFrEF population [24, 25].

The above promising findings of recently conducted trials imply that the complementary approach with SGLT2i as an added advantage should be considered in HFrEF patients, both diabetic and non-diabetic. Due to clinical benefits, observed shortly after the implementation of the therapy, the authors believe that HFrEF treatment with this group of drugs should be started early. However, their therapeutic role regards as many as three conditions, i.e. diabetes, heart failure and chronic kidney disease, and this combination seems to be a vicious circle.

## Omecamtiv mecarbil—a novel hope for patients with HFrEF?

This molecule has been known for several years, in particular since findings of the phase 2 trial COSMIC-HF (Chronic Oral Study of Myosin Activation to Increase Contractility in Heart Failure) were published [26]. The mechanism of omecamtiv mecarbil action is completely different from that observed in other therapies and unrelated to neuromodulation. Omecamtiv mecarbil is a cardiac myosin activator representing a novel class of myotropes. The study population of COSMIC-HF consisted of 448 patients in functional class II or III, with elevated NT-proBNP and LVEF 40% or less. It has been documented that participants treated with omecamtiv mecarbil compared with those who were administered placebo demonstrated improved cardiac performance and structure in a median 20-week follow-up, which was manifested by reduced LV systolic and diastolic dimensions, which in turn resulted in an increase in the stroke volume and systolic ejection time.

In contrast to COSMIC-HF, the GALACTIC-HF trial (Global Approach to Lowering Adverse Cardiac Outcomes Through Improving Contractility in Heart Failure) was designed in a much greater population to check if implementation of omecamtiv mecarbil substantially reduces the risk of CV deaths, prevent HF episodes and improves the quality of life of HFrEF patients. The primary end point was served to estimate the time to an occurrence of cardiovascular death or first HF event (hospitalization or urgent visit due to HF) [27]. This study included over 8200 patients (25% were inpatients) with HFrEF with EF 35% or less, who were randomly assigned to receive omecamtiv mecarbil twice daily or placebo within a median 21.8-month follow-up. It should be noted that the study included patients with more advanced symptoms of HF compared with other clinical trials designed in HFrEF population—about 47% of them were in III–IV NYHA, with mean LVEF 26.6%. Over a third of the participants have been hospitalized within the last 3 months. Moreover, 65% of them were well treated with standard HFrEF therapy (19.3% were taking ARNI) and almost half of the study group had cardiac devices (cardiac resynchronization therapy and implantable cardioverter defibrillator). What is unique and distinguishing in the study population is the fact that the patients included in this study were usually excluded from other heart failure studies—with eGFR ≥ 20–30 ml/min/1.73 m^2^ (6.4% of the total enrolment; not dialyzed) and SBP (systolic blood pressure) ≥ 85 mmHg (13.7%) [28]. This is absolutely the first trial designed to check the effect and safety of selective, direct increase in cardiac contractility with omecamtiv mecarbil on serious CV outcomes in HFrEF patients. It has been documented that patients (both inpatient and outpatients), treated with omecamtiv mecarbil, had a lower risk of the primary outcome composed of first HF event or death from CV causes than those who received placebo (HR, 0.92; CI, 0.86–0.99; *P* = 0.03) [29]. What is worth emphasizing is the fact that the beneficial effect was greater in more symptomatic patients with LVEF less than 28%. The results for secondary end-points were surprising and somewhat disappointing – it was not shown that omecamtiv mecarbil significantly reduces the occurrence of CV death, first hospitalization for HF and death from any cause evaluated separately.

## Vericiguat—victory in the fight against HFrEF?

The nitric oxide (NO)—soluble guanylate cyclase (sGC)—cyclic guanosine monophosphate (cGMP) pathway is a subject of scientific interest as it might be a potential therapeutic target for patients with symptomatic HF. It plays an essential role in regulating the cardiac and endothelial function. In HFrEF population, a dysfunction of NO-sGC-cGMP signalling is observed, and it is secondary to reduced NO bioavailability. Pathophysiological facts confirm that direct activation of sGC suggests it will be a promising therapeutic approach [30]

Vericiguat is a novel oral sGC stimulator enhancing cGMP. Findings of the SOCRATES-REDUCED trial (pha.se 2b) revealed that administration of vericiguat in high-risk patients (with worsening HF after clinical stabilization) was safe and resulted in a dose-dependent reduction of NT-proBNP concentration in a short 12-week follow-up [31]. The VICTORIA trial (Vericiguat Global Study in Subjects with Heart Failure with Reduced Ejection Fraction) was designed to investigate the efficacy and safety of vericiguat in patients with chronic HFrEF with EF 45% or less (85.8% of individuals with EF < 40%), with recent worsening HF requiring a hospitalization or an intravenous diuretic therapy [32, 33]. Finally, 5050 participants were included and randomly assigned to the vericiguat group (target dose up-titrated to 10 mg once daily) or to the placebo group. It is noteworthy that the study group included patients with a serious impairment of the renal function with the eGFR level as low as 15 ml/min/1.73 m^2^ (15% of the total number of patients demonstrated eGFR 15–30 ml/min/1.73 m^2^). Moreover, 41% of randomized patients were more symptomatic, i.e., in advanced classes: III or IV NYHA, but mostly, they were well treated with optimal HFrEF pharmacotherapy. Compared with study populations of PARADIGM-HF, DAPA-HF and EMPEROR-Reduced, VICTORIA patients demonstrated more advanced HF, and two-thirds of cases were in a vulnerable period of 3 months after worsening HF symptoms. Moreover, a vast majority of VICTORIA participants (84%) were hospitalized due to HF within 6 months (Table 1). The composite primary outcome (death due to cardiovascular reasons or first hospitalization due to HF) was less frequently observed in the intervention group compared with the placebo group in a median 10.8-month follow-up (HR, 0.90; CI, 0.82–98, *P* > 0.02). What is noteworthy is the fact that beneficial clinical effects have been confirmed for patients with concentration of NT-proBNP up to 8000 pg/ml [34]. A difference in favour of vericiguat was noted after a 3-month observation and was observed throughout the study. This several-month time needed to achieve beneficial result suggests that vericiguat will not become a first-line drug in HFrEF treatment but can be an effective therapeutic option for high-risk HFrEF patients with previous episodes of worsening HF.

The results of VICTORIA trial have established the role of vericiguat in HFrEF patients. In early 2021, US FDA has approved the first sGC stimulator for HFrEF population with EF less than 45% to reduce the risk of CV death and HF hospitalization [35]. Therefore, we are witnessing another breakthrough in HFrEF treatment.

## It is time for new algorithms for HFrEF patients including novel therapies

By basing on recent SGLT2i trial results, Rosano et al. [36] and McMurray and al. [37] have proposed completely new algorithms in HFrEF management. After receiving results of DAPA-HF and EMPEROR-Reduced, symptomatic patients with HFrEF should implement the SGLT2i therapy as soon as possible. Of many drugs, dapagliflozin is preferred, and SGLT2i can be included in any stage of disease irrespective of current pharmacological treatment. According to Rosano et al., the other disease-modifying agents—ACE inhibitors/ARBs/ARNIs, beta-blockers and MRAs (mineralocorticoid receptor antagonists)—are administered in the same treatment stages as it was recommended in the 2016 guidelines. McMurray et al. go one step forwards. They propose a novel approach to HFrEF which covers three following stages: (1) beta-blocker + SGLT2i, (2) ARNI and (3) MRA—all with up-titration to target doses within 4 weeks [37]. This scheme makes it possible to implement an adequate therapy which will effectively reduce the risk of hospitalization and death in patients with HFrEF in a fairly short period of time. Particular consideration will be given to SGLT2i, which is supposed to make up foundations for new recommendations for HFrEF treatment.

Considering the results of recent HFrEF trials, the experts of the American College of Cardiology have also proposed an update for HFrEF management [38]. They suggest that the first-line treatment in symptomatic patients should be started with ACEI/ARB/ARNI (preferred ARNI), a beta-blocker, and diuretics if needed. SGLT2i occupies the second step of this algorithm, the same as e.g. MRA or ivabradine.

Bauersachs, in his editorial comment in the European Heart Journal, issued in early 2021, pointed out that the fantastic four, i.e. the combined therapy of ARNI, SGLT2i, beta-blocker and MRA, should be the main target in HFrEF [39]. Considering various clinical circumstances and heterogeneity of HFrEF population, other forms of pharmacotherapy and electrotherapy are waiting to be implemented.

**Fig. 4:**
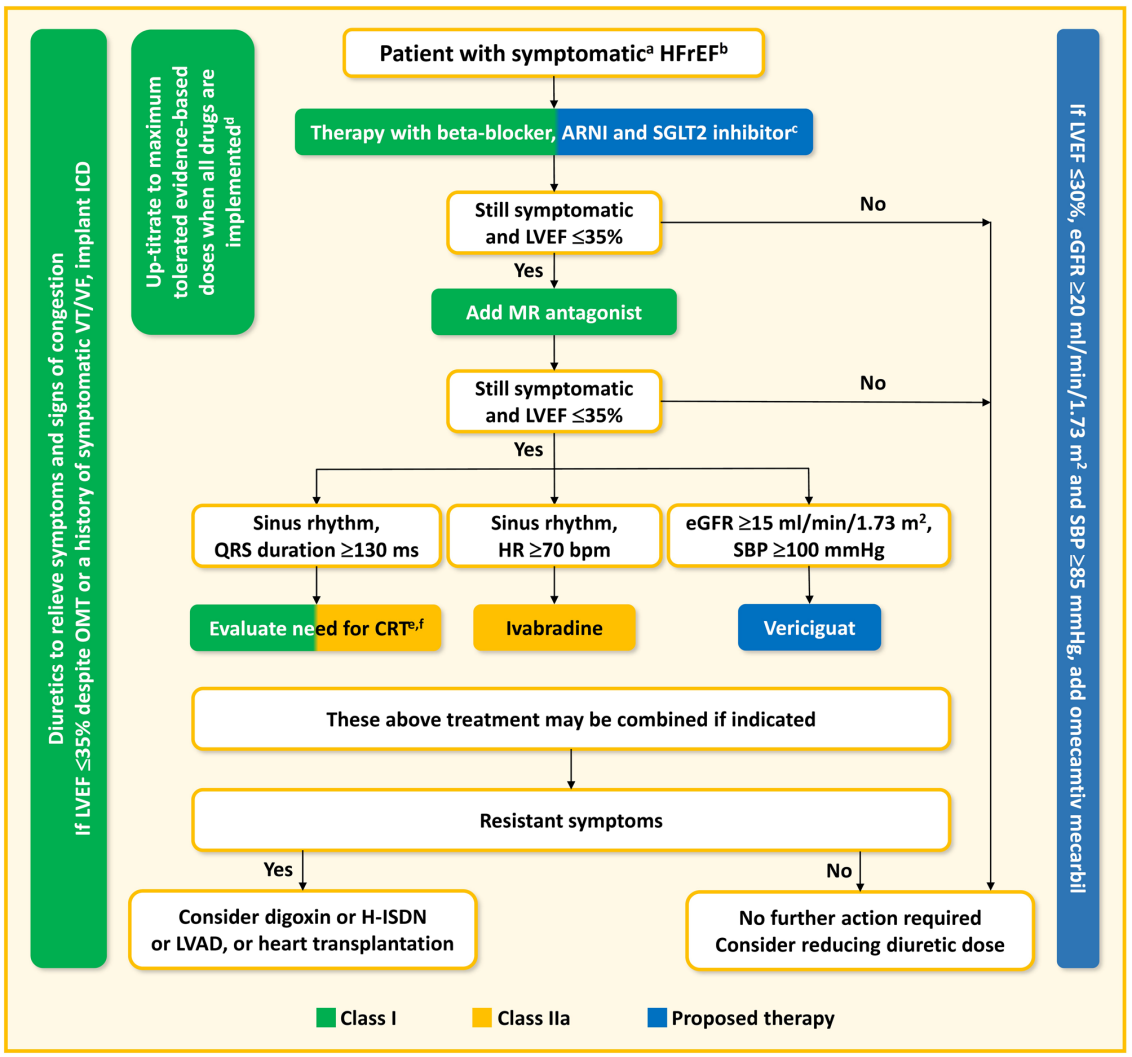
Suggests therapeutic algorithm for a patient with symptomatic heart failure with reduced ejection fraction including administration of novel drugs. Green indicates a class I recommendation; yellow indicates a class IIa recommendation; blue indicates proposed therapy (4, 36, 37). ^a^NYHA class II-IV, ^b^LVEF < 40%, ^c^beta-blocker is recommended, ^d^if not contraindicated, ^e^CRT is recommended if QRS ≥ 130 ms and LBBB (in sinus rhythm), ^f^CRT should/may be considered if QRS ≥ 130 ms with non-LBBB (in a sinus rhythm) or for patients in AF provided a strategy to ensure bi-ventricular pacing. AF atrial fibrillation, ARNI angiotensin receptor/neprilysin inhibitor, bpm beats per minute, CRT cardiac resynchronization therapy, eGFR estimated glomerular filtration rate, HFrEF heart failure with reduced ejection fraction, H-ISDN hydralazine–isosorbide dinitrate, HR heart rate, ICD implantable cardioverter-defibrillator, LBBB left bundle branch block, LVAD left ventricle assist device, LVEF left ventricle ejection fraction, MR mineralocorticoid receptor, NYHA New York Heart Association, OMT optimal medical therapy, SBP systolic blood pressure, SGLT2 sodium-glucose co-transporter-2, VT/VF ventricular tachycardia/ventricular fibrillation

Where is the place in these diagrams for absolutely new molecules such as omecamtiv mecarbil and vericiguat? The armamentarium of HFrEF therapy has significantly expanded and can also be modified as it is presented by authors on Fig. 4, with respect to ARNI, also being a first-line HFrEF treatment. The authors of this manuscript wish to emphasize the role of ARNI in modulation of the renin angiotensin aldosterone system, which is fundamental in pathophysiological pathways in HFrEF. We would like to draw attention to the possibility of using omecamtiv mecarbil as a muscle activator at every treatment stage in patients with HFrEF, in particular those with LVEF less than 30%, paying attention to the unusual possibility of using the drug in patients with SBP just over 85 mmHg and with eGFR as low as 20 ml/min/1.73 m^2^.

## Conclusions

The progressive nature of HFrEF undoubtedly requires a multifactorial intervention. The recent research data with proven drug therapies offer new and effective treatment options for patients with HFrEF which can be additionally flexible and individualized. There are indications that the landscape of contemporary heart failure is changing.
